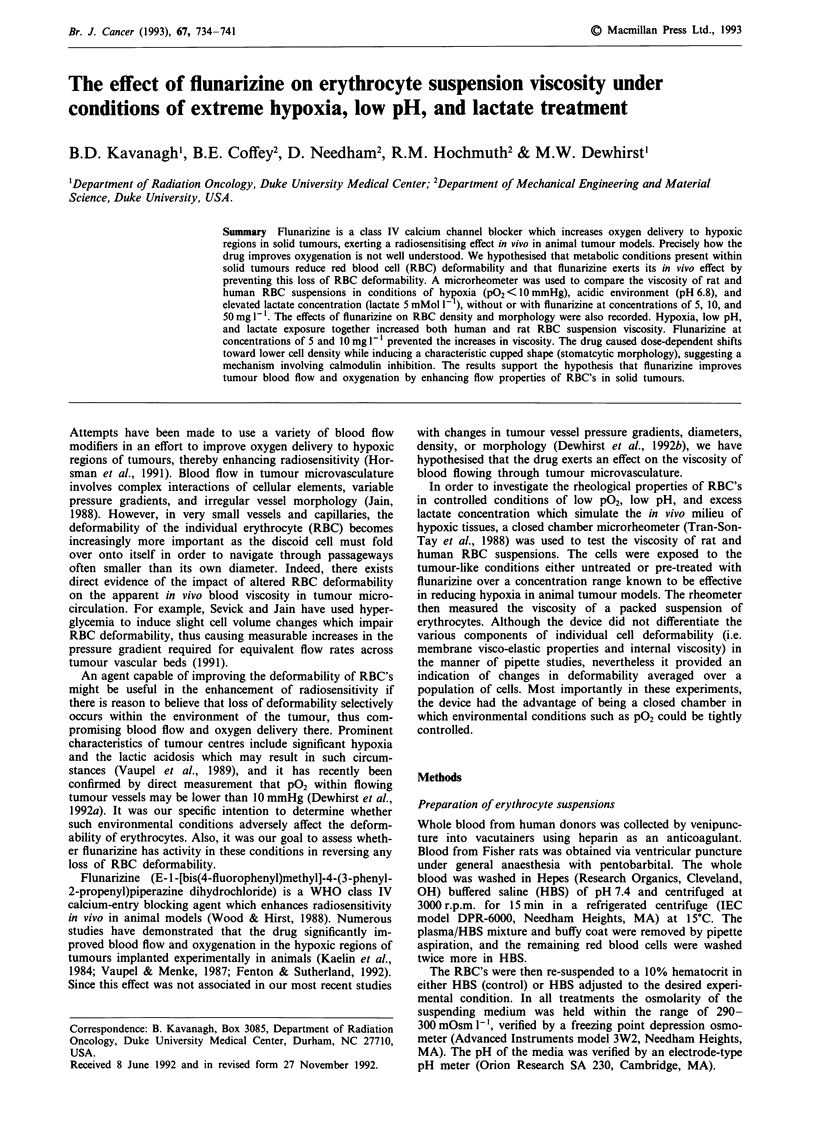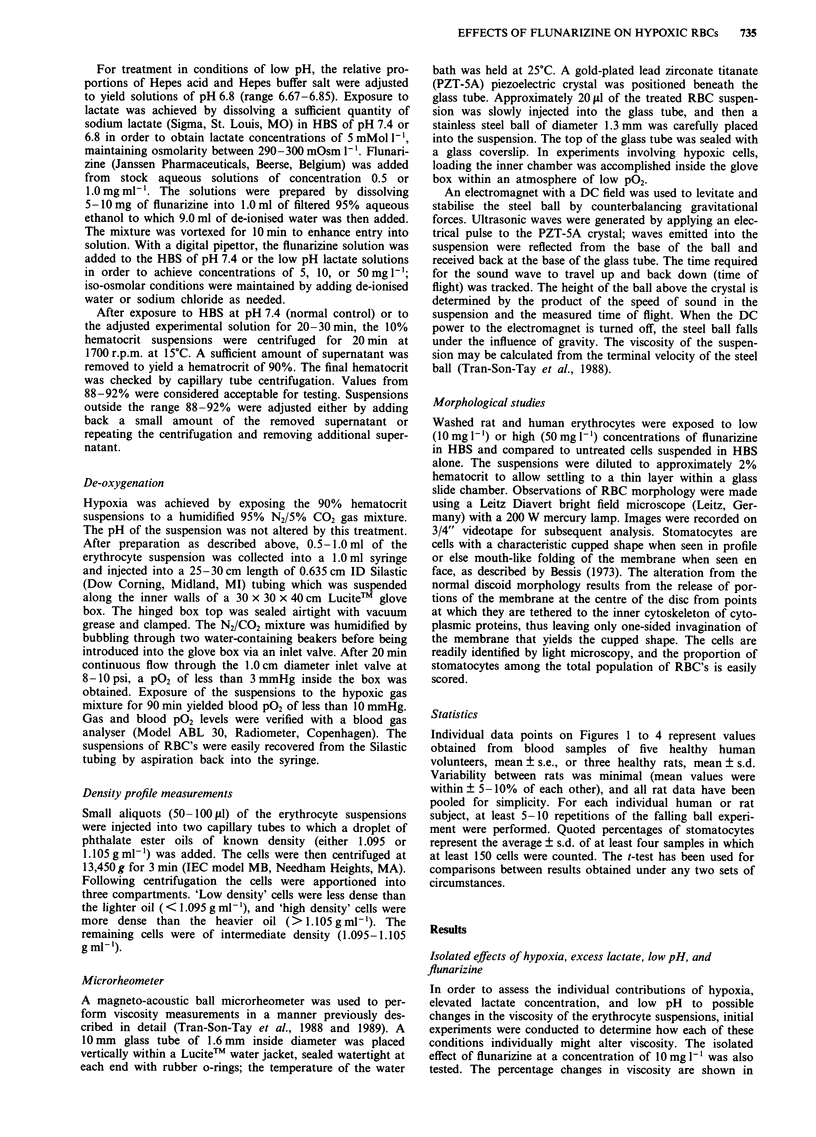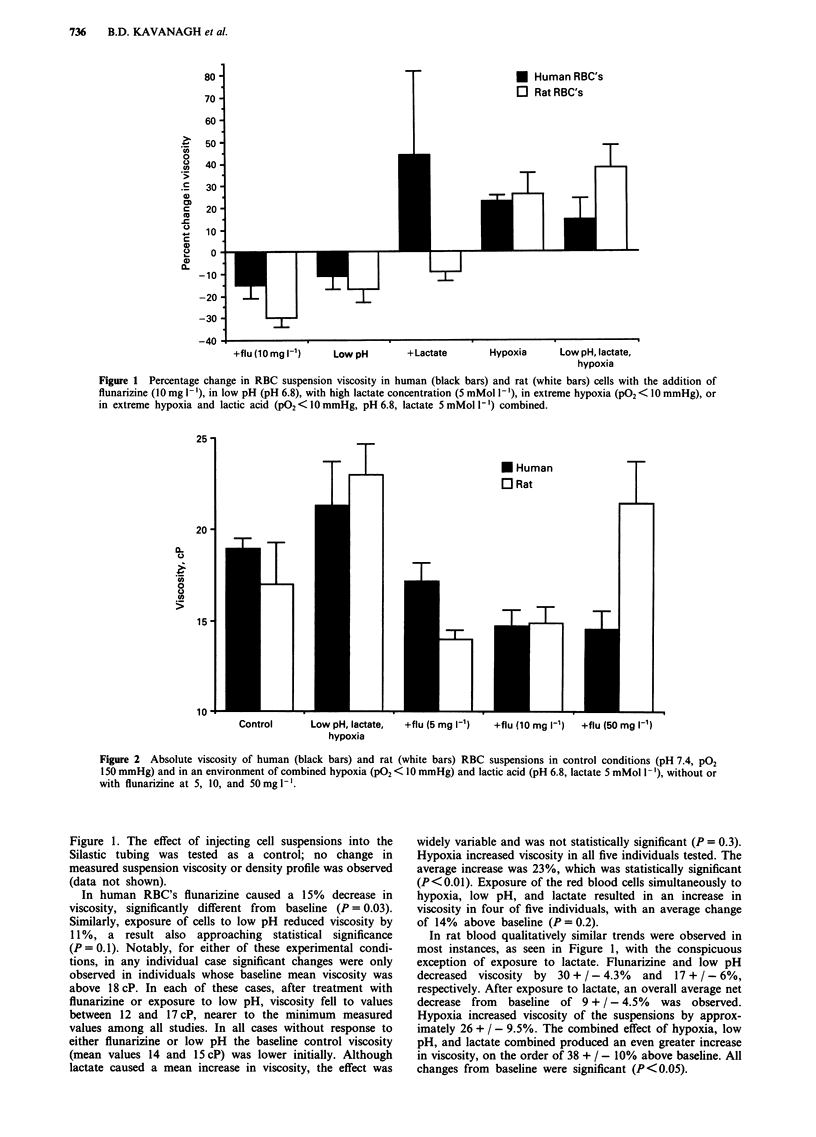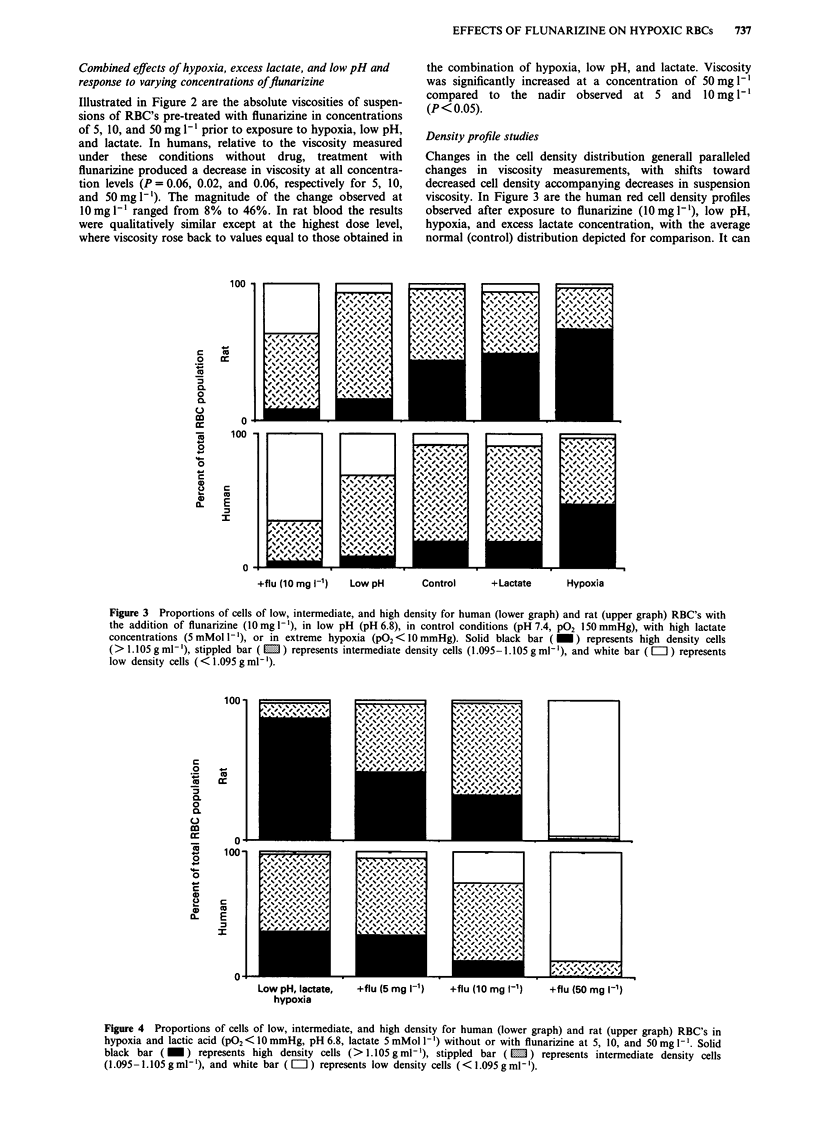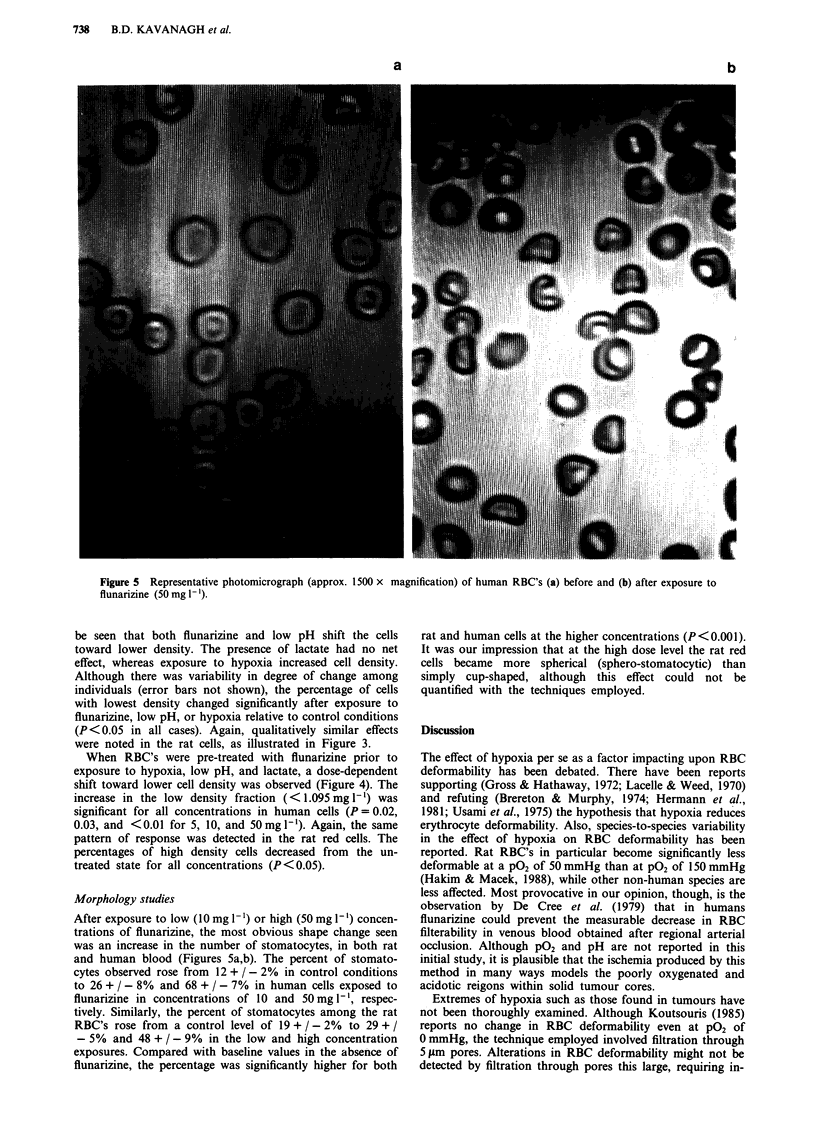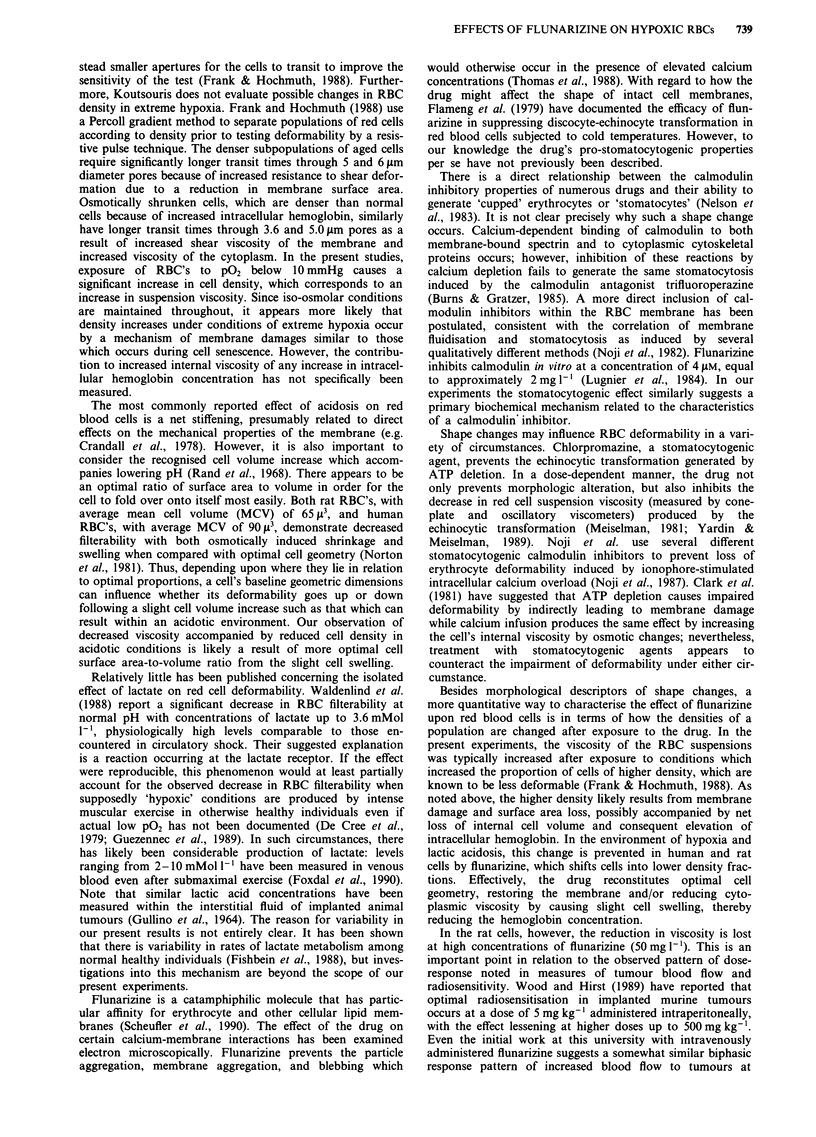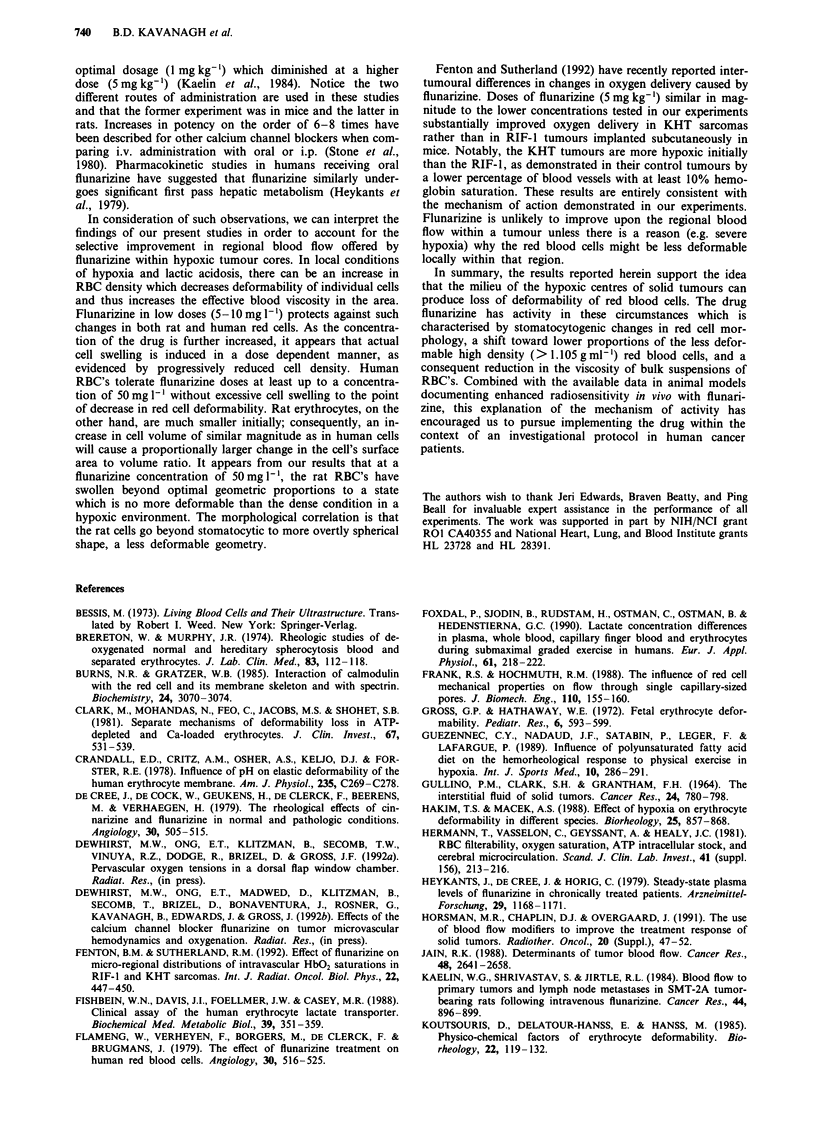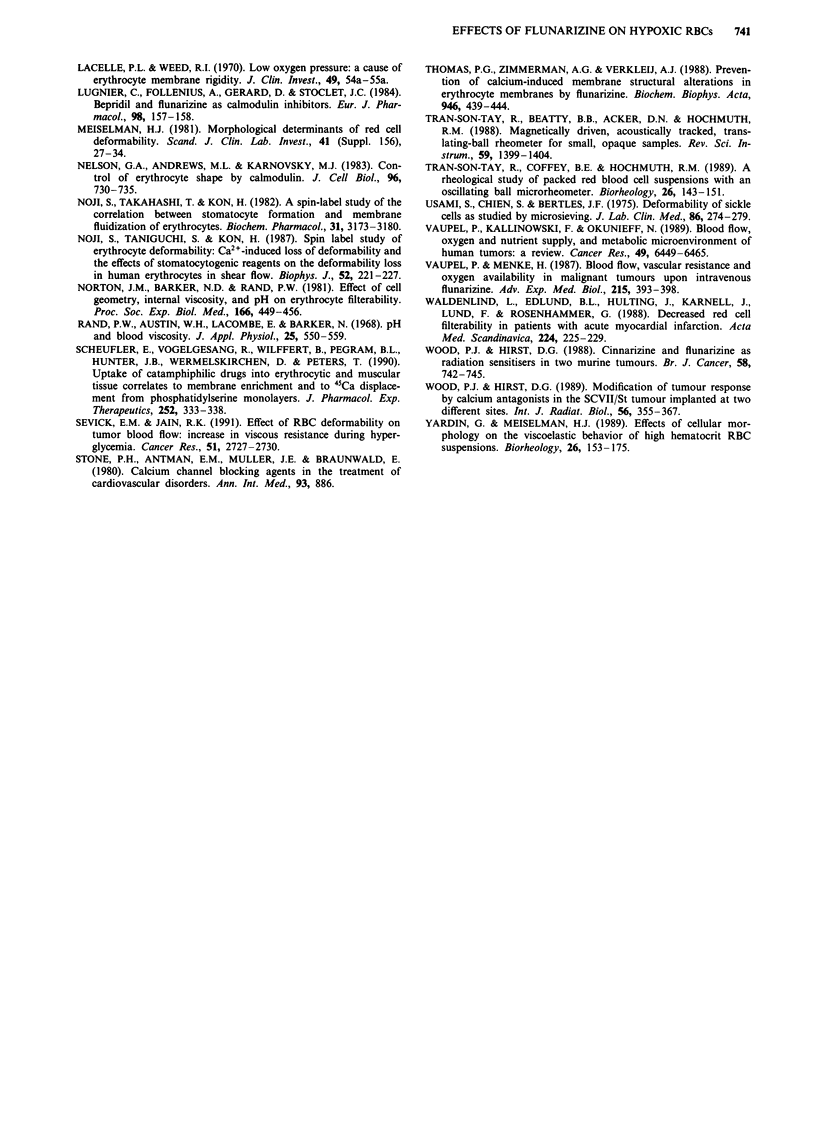# The effect of flunarizine on erythrocyte suspension viscosity under conditions of extreme hypoxia, low pH, and lactate treatment.

**DOI:** 10.1038/bjc.1993.134

**Published:** 1993-04

**Authors:** B. D. Kavanagh, B. E. Coffey, D. Needham, R. M. Hochmuth, M. W. Dewhirst

**Affiliations:** Department of Radiation Oncology, Duke University Medical Center, Durham, NC 27710.

## Abstract

**Images:**


Br. J. Cancer (1993), 67, 734–741 © Macmillan Press Ltd., 1993

### The effect of flunarizine on erythrocyte suspension viscosity under conditions of extreme hypoxia, low pH, and lactate treatment

B.D. Kavanagh[1], B.E. Coffey[2], D. Needham[2], R.M. Hochmuth[2] & M.W. Dewhirst[1]

[1]*Department of Radiation Oncology, Duke University Medical Center;* [2]*Department of Mechanical Engineering and Material Science, Duke University, USA.*

**Summary** Flunarizine is a class IV calcium channel blocker which increases oxygen delivery to hypoxic regions in solid tumours, exerting a radiosensitising effect *in vivo* in animal tumour models. Precisely how the drug improves oxygenation is not well understood. We hypothesised that metabolic conditions present within solid tumours reduce red blood cell (RBC) deformability and that flunarizine exerts its *in vivo* effect by preventing this loss of RBC deformability. A microrheometer was used to compare the viscosity of rat and human RBC suspensions in conditions of hypoxia ($pO_2 < 10$ mmHg), acidic environment (pH 6.8), and elevated lactate concentration (lactate 5 mMol $l^{-1}$), without or with flunarizine at concentrations of 5, 10, and 50 mg $l^{-1}$. The effects of flunarizine on RBC density and morphology were also recorded. Hypoxia, low pH, and lactate exposure together increased both human and rat RBC suspension viscosity. Flunarizine at concentrations of 5 and 10 mg $l^{-1}$ prevented the increases in viscosity. The drug caused dose-dependent shifts toward lower cell density while inducing a characteristic cupped shape (stomatcytic morphology), suggesting a mechanism involving calmodulin inhibition. The results support the hypothesis that flunarizine improves tumour blood flow and oxygenation by enhancing flow properties of RBC's in solid tumours.

Attempts have been made to use a variety of blood flow modifiers in an effort to improve oxygen delivery to hypoxic regions of tumours, thereby enhancing radiosensitivity (Horsman *et al.*, 1991). Blood flow in tumour microvasculature involves complex interactions of cellular elements, variable pressure gradients, and irregular vessel morphology (Jain, 1988). However, in very small vessels and capillaries, the deformability of the individual erythrocyte (RBC) becomes increasingly more important as the discoid cell must fold over onto itself in order to navigate through passageways often smaller than its own diameter. Indeed, there exists direct evidence of the impact of altered RBC deformability on the apparent *in vivo* blood viscosity in tumour microcirculation. For example, Sevick and Jain have used hyperglycemia to induce slight cell volume changes which impair RBC deformability, thus causing measurable increases in the pressure gradient required for equivalent flow rates across tumour vascular beds (1991).

An agent capable of improving the deformability of RBC's might be useful in the enhancement of radiosensitivity if there is reason to believe that loss of deformability selectively occurs within the environment of the tumour, thus compromising blood flow and oxygen delivery there. Prominent characteristics of tumour centres include significant hypoxia and the lactic acidosis which may result in such circumstances (Vaupel *et al.*, 1989), and it has recently been confirmed by direct measurement that $pO_2$ within flowing tumour vessels may be lower than 10 mmHg (Dewhirst *et al.*, 1992*a*). It was our specific intention to determine whether such environmental conditions adversely affect the deformability of erythrocytes. Also, it was our goal to assess whether flunarizine has activity in these conditions in reversing any loss of RBC deformability.

Flunarizine (E-1-[bis(4-fluorophenyl)methyl]-4-(3-phenyl-2-propenyl)piperazine dihydrochloride) is a WHO class IV calcium-entry blocking agent which enhances radiosensitivity *in vivo* in animal models (Wood & Hirst, 1988). Numerous studies have demonstrated that the drug significantly improved blood flow and oxygenation in the hypoxic regions of tumours implanted experimentally in animals (Kaelin *et al.*, 1984; Vaupel & Menke, 1987; Fenton & Sutherland, 1992). Since this effect was not associated in our most recent studies with changes in tumour vessel pressure gradients, diameters, density, or morphology (Dewhirst *et al.*, 1992*b*), we have hypothesised that the drug exerts an effect on the viscosity of blood flowing through tumour microvasculature.

In order to investigate the rheological properties of RBC's in controlled conditions of low $pO_2$, low pH, and excess lactate concentration which simulate the *in vivo* milieu of hypoxic tissues, a closed chamber microrheometer (Tran-Son-Tay *et al.*, 1988) was used to test the viscosity of rat and human RBC suspensions. The cells were exposed to the tumour-like conditions either untreated or pre-treated with flunarizine over a concentration range known to be effective in reducing hypoxia in animal tumour models. The rheometer then measured the viscosity of a packed suspension of erythrocytes. Although the device did not differentiate the various components of individual cell deformability (i.e. membrane visco-elastic properties and internal viscosity) in the manner of pipette studies, nevertheless it provided an indication of changes in deformability averaged over a population of cells. Most importantly in these experiments, the device had the advantage of being a closed chamber in which environmental conditions such as $pO_2$ could be tightly controlled.

### Methods

### *Preparation of erythrocyte suspensions*

Whole blood from human donors was collected by venipuncture into vacutainers using heparin as an anticoagulant. Blood from Fisher rats was obtained via ventricular puncture under general anaesthesia with pentobarbital. The whole blood was washed in Hepes (Research Organics, Cleveland, OH) buffered saline (HBS) of pH 7.4 and centrifuged at 3000 r.p.m. for 15 min in a refrigerated centrifuge (IEC model DPR-6000, Needham Heights, MA) at 15°C. The plasma/HBS mixture and buffy coat were removed by pipette aspiration, and the remaining red blood cells were washed twice more in HBS.

The RBC's were then re-suspended to a 10% hematocrit in either HBS (control) or HBS adjusted to the desired experimental condition. In all treatments the osmolarity of the suspending medium was held within the range of 290–300 mOsm $l^{-1}$, verified by a freezing point depression osmometer (Advanced Instruments model 3W2, Needham Heights, MA). The pH of the media was verified by an electrode-type pH meter (Orion Research SA 230, Cambridge, MA).

Correspondence: B. Kavanagh, Box 3085, Department of Radiation Oncology, Duke University Medical Center, Durham, NC 27710, USA.
Received 8 June 1992 and in revised form 27 November 1992.

For treatment in conditions of low pH, the relative proportions of Hepes acid and Hepes buffer salt were adjusted to yield solutions of pH 6.8 (range 6.67–6.85). Exposure to lactate was achieved by dissolving a sufficient quantity of sodium lactate (Sigma, St. Louis, MO) in HBS of pH 7.4 or 6.8 in order to obtain lactate concentrations of 5 mMol $l^{-1}$, maintaining osmolarity between 290–300 mOsm $l^{-1}$. Flunarizine (Janssen Pharmaceuticals, Beerse, Belgium) was added from stock aqueous solutions of concentration 0.5 or 1.0 mg $ml^{-1}$. The solutions were prepared by dissolving 5–10 mg of flunarizine into 1.0 ml of filtered 95% aqueous ethanol to which 9.0 ml of de-ionised water was then added. The mixture was vortexed for 10 min to enhance entry into solution. With a digital pipettor, the flunarizine solution was added to the HBS of pH 7.4 or the low pH lactate solutions in order to achieve concentrations of 5, 10, or 50 mg $l^{-1}$; iso-osmolar conditions were maintained by adding de-ionised water or sodium chloride as needed.

After exposure to HBS at pH 7.4 (normal control) or to the adjusted experimental solution for 20–30 min, the 10% hematocrit suspensions were centrifuged for 20 min at 1700 r.p.m. at 15°C. A sufficient amount of supernatant was removed to yield a hematrocrit of 90%. The final hematocrit was checked by capillary tube centrifugation. Values from 88–92% were considered acceptable for testing. Suspensions outside the range 88–92% were adjusted either by adding back a small amount of the removed supernatant or repeating the centrifugation and removing additional supernatant.

### *De-oxygenation*

Hypoxia was achieved by exposing the 90% hematocrit suspensions to a humidified 95% $N_2$/5% $CO_2$ gas mixture. The pH of the suspension was not altered by this treatment. After preparation as described above, 0.5–1.0 ml of the erythrocyte suspension was collected into a 1.0 ml syringe and injected into a 25–30 cm length of 0.635 cm ID Silastic (Dow Corning, Midland, MI) tubing which was suspended along the inner walls of a 30 × 30 × 40 cm Lucite™ glove box. The hinged box top was sealed airtight with vacuum grease and clamped. The $N_2$/$CO_2$ mixture was humidified by bubbling through two water-containing beakers before being introduced into the glove box via an inlet valve. After 20 min continuous flow through the 1.0 cm diameter inlet valve at 8–10 psi, a $pO_2$ of less than 3 mmHg inside the box was obtained. Exposure of the suspensions to the hypoxic gas mixture for 90 min yielded blood $pO_2$ of less than 10 mmHg. Gas and blood $pO_2$ levels were verified with a blood gas analyser (Model ABL 30, Radiometer, Copenhagen). The suspensions of RBC's were easily recovered from the Silastic tubing by aspiration back into the syringe.

### *Density profile measurements*

Small aliquots (50–100 μl) of the erythrocyte suspensions were injected into two capillary tubes to which a droplet of phthalate ester oils of known density (either 1.095 or 1.105 g $ml^{-1}$) was added. The cells were then centrifuged at 13,450 ***g*** for 3 min (IEC model MB, Needham Heights, MA). Following centrifugation the cells were apportioned into three compartments. 'Low density' cells were less dense than the lighter oil ($<$1.095 g $ml^{-1}$), and 'high density' cells were more dense than the heavier oil ($>$1.105 g $ml^{-1}$). The remaining cells were of intermediate density (1.095–1.105 g $ml^{-1}$).

### *Microrheometer*

A magneto-acoustic ball microrheometer was used to perform viscosity measurements in a manner previously described in detail (Tran-Son-Tay *et al.*, 1988 and 1989). A 10 mm glass tube of 1.6 mm inside diameter was placed vertically within a Lucite™ water jacket, sealed watertight at each end with rubber o-rings; the temperature of the water bath was held at 25°C. A gold-plated lead zirconate titanate (PZT-5A) piezoelectric crystal was positioned beneath the glass tube. Approximately 20 μl of the treated RBC suspension was slowly injected into the glass tube, and then a stainless steel ball of diameter 1.3 mm was carefully placed into the suspension. The top of the glass tube was sealed with a glass coverslip. In experiments involving hypoxic cells, loading the inner chamber was accomplished inside the glove box within an atmosphere of low $pO_2$.

An electromagnet with a DC field was used to levitate and stabilise the steel ball by counterbalancing gravitational forces. Ultrasonic waves were generated by applying an electrical pulse to the PZT-5A crystal; waves emitted into the suspension were reflected from the base of the ball and received back at the base of the glass tube. The time required for the sound wave to travel up and back down (time of flight) was tracked. The height of the ball above the crystal is determined by the product of the speed of sound in the suspension and the measured time of flight. When the DC power to the electromagnet is turned off, the steel ball falls under the influence of gravity. The viscosity of the suspension may be calculated from the terminal velocity of the steel ball (Tran-Son-Tay *et al.*, 1988).

### *Morphological studies*

Washed rat and human erythrocytes were exposed to low (10 mg $l^{-1}$) or high (50 mg $l^{-1}$) concentrations of flunarizine in HBS and compared to untreated cells suspended in HBS alone. The suspensions were diluted to approximately 2% hematocrit to allow settling to a thin layer within a glass slide chamber. Observations of RBC morphology were made using a Leitz Diavert bright field microscope (Leitz, Germany) with a 200 W mercury lamp. Images were recorded on 3/4″ videotape for subsequent analysis. Stomatocytes are cells with a characteristic cupped shape when seen in profile or else mouth-like folding of the membrane when seen en face, as described by Bessis (1973). The alteration from the normal discoid morphology results from the release of portions of the membrane at the centre of the disc from points at which they are tethered to the inner cytoskeleton of cytoplasmic proteins, thus leaving only one-sided invagination of the membrane that yields the cupped shape. The cells are readily identified by light microscopy, and the proportion of stomatocytes among the total population of RBC's is easily scored.

### *Statistics*

Individual data points on Figures 1 to 4 represent values obtained from blood samples of five healthy human volunteers, mean ± s.e., or three healthy rats, mean ± s.d. Variability between rats was minimal (mean values were within ± 5–10% of each other), and all rat data have been pooled for simplicity. For each individual human or rat subject, at least 5–10 repetitions of the falling ball experiment were performed. Quoted percentages of stomatocytes represent the average ± s.d. of at least four samples in which at least 150 cells were counted. The *t*-test has been used for comparisons between results obtained under any two sets of circumstances.

### Results

### *Isolated effects of hypoxia, excess lactate, low pH, and flunarizine*

In order to assess the individual contributions of hypoxia, elevated lactate concentration, and low pH to possible changes in the viscosity of the erythrocyte suspensions, initial experiments were conducted to determine how each of these conditions individually might alter viscosity. The isolated effect of flunarizine at a concentration of 10 mg $l^{-1}$ was also tested. The percentage changes in viscosity are shown in

**Figure 1** Percentage change in RBC suspension viscosity in human (black bars) and rat (white bars) cells with the addition of flunarizine ($10\,mg\,l^{-1}$), in low pH (pH 6.8), with high lactate concentration ($5\,mMol\,l^{-1}$), in extreme hypoxia ($pO_2 < 10$ mmHg), or in extreme hypoxia and lactic acid ($pO_2 < 10$ mmHg, pH 6.8, lactate $5\,mMol\,l^{-1}$) combined.

**Figure 2** Absolute viscosity of human (black bars) and rat (white bars) RBC suspensions in control conditions (pH 7.4, $pO_2$ 150 mmHg) and in an environment of combined hypoxia ($pO_2 < 10$ mmHg) and lactic acid (pH 6.8, lactate $5\,mMol\,l^{-1}$), without or with flunarizine at 5, 10, and $50\,mg\,l^{-1}$.

Figure 1. The effect of injecting cell suspensions into the Silastic tubing was tested as a control; no change in measured suspension viscosity or density profile was observed (data not shown).

In human RBC's flunarizine caused a 15% decrease in viscosity, significantly different from baseline ($P = 0.03$). Similarly, exposure of cells to low pH reduced viscosity by 11%, a result also approaching statistical significance ($P = 0.1$). Notably, for either of these experimental conditions, in any individual case significant changes were only observed in individuals whose baseline mean viscosity was above 18 cP. In each of these cases, after treatment with flunarizine or exposure to low pH, viscosity fell to values between 12 and 17 cP, nearer to the minimum measured values among all studies. In all cases without response to either flunarizine or low pH the baseline control viscosity (mean values 14 and 15 cP) was lower initially. Although lactate caused a mean increase in viscosity, the effect was widely variable and was not statistically significant ($P = 0.3$). Hypoxia increased viscosity in all five individuals tested. The average increase was 23%, which was statistically significant ($P < 0.01$). Exposure of the red blood cells simultaneously to hypoxia, low pH, and lactate resulted in an increase in viscosity in four of five individuals, with an average change of 14% above baseline ($P = 0.2$).

In rat blood qualitatively similar trends were observed in most instances, as seen in Figure 1, with the conspicuous exception of exposure to lactate. Flunarizine and low pH decreased viscosity by 30 + / − 4.3% and 17 + / − 6%, respectively. After exposure to lactate, an overall average net decrease from baseline of 9 + / − 4.5% was observed. Hypoxia increased viscosity of the suspensions by approximately 26 + / − 9.5%. The combined effect of hypoxia, low pH, and lactate combined produced an even greater increase in viscosity, on the order of 38 + / − 10% above baseline. All changes from baseline were significant ($P < 0.05$).

*Combined effects of hypoxia, excess lactate, and low pH and response to varying concentrations of flunarizine*

Illustrated in Figure 2 are the absolute viscosities of suspensions of RBC's pre-treated with flunarizine in concentrations of 5, 10, and 50 mg $l^{-1}$ prior to exposure to hypoxia, low pH, and lactate. In humans, relative to the viscosity measured under these conditions without drug, treatment with flunarizine produced a decrease in viscosity at all concentration levels ($P = 0.06$, 0.02, and 0.06, respectively for 5, 10, and 50 mg $l^{-1}$). The magnitude of the change observed at 10 mg $l^{-1}$ ranged from 8% to 46%. In rat blood the results were qualitatively similar except at the highest dose level, where viscosity rose back to values equal to those obtained in the combination of hypoxia, low pH, and lactate. Viscosity was significantly increased at a concentration of 50 mg $l^{-1}$ compared to the nadir observed at 5 and 10 mg $l^{-1}$ ($P < 0.05$).

*Density profile studies*

Changes in the cell density distribution generall paralleled changes in viscosity measurements, with shifts toward decreased cell density accompanying decreases in suspension viscosity. In Figure 3 are the human red cell density profiles observed after exposure to flunarizine (10 mg $l^{-1}$), low pH, hypoxia, and excess lactate concentration, with the average normal (control) distribution depicted for comparison. It can

**Figure 3** Proportions of cells of low, intermediate, and high density for human (lower graph) and rat (upper graph) RBC's with the addition of flunarizine (10 mg $l^{-1}$), in low pH (pH 6.8), in control conditions (pH 7.4, $pO_2$ 150 mmHg), with high lactate concentrations (5 mMol $l^{-1}$), or in extreme hypoxia ($pO_2 < 10$ mmHg). Solid black bar (■) represents high density cells ($> 1.105$ g $ml^{-1}$), stippled bar (▩) represents intermediate density cells (1.095–1.105 g $ml^{-1}$), and white bar (□) represents low density cells ($< 1.095$ g $ml^{-1}$).

**Figure 4** Proportions of cells of low, intermediate, and high density for human (lower graph) and rat (upper graph) RBC's in hypoxia and lactic acid ($pO_2 < 10$ mmHg, pH 6.8, lactate 5 mMol $l^{-1}$) without or with flunarizine at 5, 10, and 50 mg $l^{-1}$. Solid black bar (■) represents high density cells ($> 1.105$ g $ml^{-1}$), stippled bar (▩) represents intermediate density cells (1.095–1.105 g $ml^{-1}$), and white bar (□) represents low density cells ($< 1.095$ g $ml^{-1}$).

**Figure 5** Representative photomicrograph (approx. 1500 × magnification) of human RBC's (**a**) before and (**b**) after exposure to flunarizine ($50\ mg\ l^{-1}$).

be seen that both flunarizine and low pH shift the cells toward lower density. The presence of lactate had no net effect, whereas exposure to hypoxia increased cell density. Although there was variability in degree of change among individuals (error bars not shown), the percentage of cells with lowest density changed significantly after exposure to flunarizine, low pH, or hypoxia relative to control conditions ($P < 0.05$ in all cases). Again, qualitatively similar effects were noted in the rat cells, as illustrated in Figure 3.

When RBC's were pre-treated with flunarizine prior to exposure to hypoxia, low pH, and lactate, a dose-dependent shift toward lower cell density was observed (Figure 4). The increase in the low density fraction ($< 1.095\ mg\ l^{-1}$) was significant for all concentrations in human cells ($P = 0.02$, 0.03, and $< 0.01$ for 5, 10, and $50\ mg\ l^{-1}$). Again, the same pattern of response was detected in the rat red cells. The percentages of high density cells decreased from the untreated state for all concentrations ($P < 0.05$).

### *Morphology studies*

After exposure to low ($10\ mg\ l^{-1}$) or high ($50\ mg\ l^{-1}$) concentrations of flunarizine, the most obvious shape change seen was an increase in the number of stomatocytes, in both rat and human blood (Figures 5a,b). The percent of stomatocytes observed rose from $12 + / - 2\%$ in control conditions to $26 + / - 8\%$ and $68 + / - 7\%$ in human cells exposed to flunarizine in concentrations of 10 and $50\ mg\ l^{-1}$, respectively. Similarly, the percent of stomatocytes among the rat RBC's rose from a control level of $19 + / - 2\%$ to $29 + / - 5\%$ and $48 + / - 9\%$ in the low and high concentration exposures. Compared with baseline values in the absence of flunarizine, the percentage was significantly higher for both rat and human cells at the higher concentrations ($P < 0.001$). It was our impression that at the high dose level the rat red cells became more spherical (sphero-stomatocytic) than simply cup-shaped, although this effect could not be quantified with the techniques employed.

### Discussion

The effect of hypoxia per se as a factor impacting upon RBC deformability has been debated. There have been reports supporting (Gross & Hathaway, 1972; Lacelle & Weed, 1970) and refuting (Brereton & Murphy, 1974; Hermann *et al.*, 1981; Usami *et al.*, 1975) the hypothesis that hypoxia reduces erythrocyte deformability. Also, species-to-species variability in the effect of hypoxia on RBC deformability has been reported. Rat RBC's in particular become significantly less deformable at a $pO_2$ of 50 mmHg than at $pO_2$ of 150 mmHg (Hakim & Macek, 1988), while other non-human species are less affected. Most provocative in our opinion, though, is the observation by De Cree *et al.* (1979) that in humans flunarizine could prevent the measurable decrease in RBC filterability in venous blood obtained after regional arterial occlusion. Although $pO_2$ and pH are not reported in this initial study, it is plausible that the ischemia produced by this method in many ways models the poorly oxygenated and acidotic reigons within solid tumour cores.

Extremes of hypoxia such as those found in tumours have not been thoroughly examined. Although Koutsouris (1985) reports no change in RBC deformability even at $pO_2$ of 0 mmHg, the technique employed involved filtration through 5 $\mu m$ pores. Alterations in RBC deformability might not be detected by filtration through pores this large, requiring instead smaller apertures for the cells to transit to improve the sensitivity of the test (Frank & Hochmuth, 1988). Furthermore, Koutsouris does not evaluate possible changes in RBC density in extreme hypoxia. Frank and Hochmuth (1988) use a Percoll gradient method to separate populations of red cells according to density prior to testing deformability by a resistive pulse technique. The denser subpopulations of aged cells require significantly longer transit times through 5 and 6 $\mu$m diameter pores because of increased resistance to shear deformation due to a reduction in membrane surface area. Osmotically shrunken cells, which are denser than normal cells because of increased intracellular hemoglobin, similarly have longer transit times through 3.6 and 5.0 $\mu$m pores as a result of increased shear viscosity of the membrane and increased viscosity of the cytoplasm. In the present studies, exposure of RBC's to $pO_2$ below 10 mmHg causes a significant increase in cell density, which corresponds to an increase in suspension viscosity. Since iso-osmolar conditions are maintained throughout, it appears more likely that density increases under conditions of extreme hypoxia occur by a mechanism of membrane damages similar to those which occurs during cell senescence. However, the contribution to increased internal viscosity of any increase in intracellular hemoglobin concentration has not specifically been measured.

The most commonly reported effect of acidosis on red blood cells is a net stiffening, presumably related to direct effects on the mechanical properties of the membrane (e.g. Crandall *et al.*, 1978). However, it is also important to consider the recognised cell volume increase which accompanies lowering pH (Rand *et al.*, 1968). There appears to be an optimal ratio of surface area to volume in order for the cell to fold over onto itself most easily. Both rat RBC's, with average mean cell volume (MCV) of 65 $\mu^3$, and human RBC's, with average MCV of 90 $\mu^3$, demonstrate decreased filterability with both osmotically induced shrinkage and swelling when compared with optimal cell geometry (Norton *et al.*, 1981). Thus, depending upon where they lie in relation to optimal proportions, a cell's baseline geometric dimensions can influence whether its deformability goes up or down following a slight cell volume increase such as that which can result within an acidotic environment. Our observation of decreased viscosity accompanied by reduced cell density in acidotic conditions is likely a result of more optimal cell surface area-to-volume ratio from the slight cell swelling.

Relatively little has been published concerning the isolated effect of lactate on red cell deformability. Waldenlind *et al.* (1988) report a significant decrease in RBC filterability at normal pH with concentrations of lactate up to 3.6 mMol $l^{-1}$, physiologically high levels comparable to those encountered in circulatory shock. Their suggested explanation is a reaction occurring at the lactate receptor. If the effect were reproducible, this phenomenon would at least partially account for the observed decrease in RBC filterability when supposedly 'hypoxic' conditions are produced by intense muscular exercise in otherwise healthy individuals even if actual low $pO_2$ has not been documented (De Cree *et al.*, 1979; Guezennec *et al.*, 1989). In such circumstances, there has likely been considerable production of lactate: levels ranging from 2–10 mMol $l^{-1}$ have been measured in venous blood even after submaximal exercise (Foxdal *et al.*, 1990). Note that similar lactic acid concentrations have been measured within the interstitial fluid of implanted animal tumours (Gullino *et al.*, 1964). The reason for variability in our present results is not entirely clear. It has been shown that there is variability in rates of lactate metabolism among normal healthy individuals (Fishbein *et al.*, 1988), but investigations into this mechanism are beyond the scope of our present experiments.

Flunarizine is a catamphiphilic molecule that has particular affinity for erythrocyte and other cellular lipid membranes (Scheufler *et al.*, 1990). The effect of the drug on certain calcium-membrane interactions has been examined electron microscopically. Flunarizine prevents the particle aggregation, membrane aggregation, and blebbing which would otherwise occur in the presence of elevated calcium concentrations (Thomas *et al.*, 1988). With regard to how the drug might affect the shape of intact cell membranes, Flameng *et al.* (1979) have documented the efficacy of flunarizine in suppressing discocyte-echinocyte transformation in red blood cells subjected to cold temperatures. However, to our knowledge the drug's pro-stomatocytogenic properties per se have not previously been described.

There is a direct relationship between the calmodulin inhibitory properties of numerous drugs and their ability to generate 'cupped' erythrocytes or 'stomatocytes' (Nelson *et al.*, 1983). It is not clear precisely why such a shape change occurs. Calcium-dependent binding of calmodulin to both membrane-bound spectrin and to cytoplasmic cytoskeletal proteins occurs; however, inhibition of these reactions by calcium depletion fails to generate the same stomatocytosis induced by the calmodulin antagonist trifluoroperazine (Burns & Gratzer, 1985). A more direct inclusion of calmodulin inhibitors within the RBC membrane has been postulated, consistent with the correlation of membrane fluidisation and stomatocytosis as induced by several qualitatively different methods (Noji *et al.*, 1982). Flunarizine inhibits calmodulin *in vitro* at a concentration of 4 $\mu$M, equal to approximately 2 mg $l^{-1}$ (Lugnier *et al.*, 1984). In our experiments the stomatocytogenic effect similarly suggests a primary biochemical mechanism related to the characteristics of a calmodulin inhibitor.

Shape changes may influence RBC deformability in a variety of circumstances. Chlorpromazine, a stomatocytogenic agent, prevents the echinocytic transformation generated by ATP deletion. In a dose-dependent manner, the drug not only prevents morphologic alteration, but also inhibits the decrease in red cell suspension viscosity (measured by cone-plate and oscillatory viscometers) produced by the echinocytic transformation (Meiselman, 1981; Yardin & Meiselman, 1989). Noji *et al.* use several different stomatocytogenic calmodulin inhibitors to prevent loss of erythrocyte deformability induced by ionophore-stimulated intracellular calcium overload (Noji *et al.*, 1987). Clark *et al.* (1981) have suggested that ATP depletion causes impaired deformability by indirectly leading to membrane damage while calcium infusion produces the same effect by increasing the cell's internal viscosity by osmotic changes; nevertheless, treatment with stomatocytogenic agents appears to counteract the impairment of deformability under either circumstance.

Besides morphological descriptors of shape changes, a more quantitative way to characterise the effect of flunarizine upon red blood cells is in terms of how the densities of a population are changed after exposure to the drug. In the present experiments, the viscosity of the RBC suspensions was typically increased after exposure to conditions which increased the proportion of cells of higher density, which are known to be less deformable (Frank & Hochmuth, 1988). As noted above, the higher density likely results from membrane damage and surface area loss, possibly accompanied by net loss of internal cell volume and consequent elevation of intracellular hemoglobin. In the environment of hypoxia and lactic acidosis, this change is prevented in human and rat cells by flunarizine, which shifts cells into lower density fractions. Effectively, the drug reconstitutes optimal cell geometry, restoring the membrane and/or reducing cytoplasmic viscosity by causing slight cell swelling, thereby reducing the hemoglobin concentration.

In the rat cells, however, the reduction in viscosity is lost at high concentrations of flunarizine (50 mg $l^{-1}$). This is an important point in relation to the observed pattern of dose-response noted in measures of tumour blood flow and radiosensitivity. Wood and Hirst (1989) have reported that optimal radiosensitisation in implanted murine tumours occurs at a dose of 5 mg $kg^{-1}$ administered intraperitoneally, with the effect lessening at higher doses up to 500 mg $kg^{-1}$. Even the initial work at this university with intravenously administered flunarizine suggests a somewhat similar biphasic response pattern of increased blood flow to tumours at optimal dosage ($1\,mg\,kg^{-1}$) which diminished at a higher dose ($5\,mg\,kg^{-1}$) (Kaelin *et al.*, 1984). Notice the two different routes of administration are used in these studies and that the former experiment was in mice and the latter in rats. Increases in potency on the order of 6–8 times have been described for other calcium channel blockers when comparing i.v. administration with oral or i.p. (Stone *et al.*, 1980). Pharmacokinetic studies in humans receiving oral flunarizine have suggested that flunarizine similarly undergoes significant first pass hepatic metabolism (Heykants *et al.*, 1979).

In consideration of such observations, we can interpret the findings of our present studies in order to account for the selective improvement in regional blood flow offered by flunarizine within hypoxic tumour cores. In local conditions of hypoxia and lactic acidosis, there can be an increase in RBC density which decreases deformability of individual cells and thus increases the effective blood viscosity in the area. Flunarizine in low doses ($5-10\,mg\,l^{-1}$) protects against such changes in both rat and human red cells. As the concentration of the drug is further increased, it appears that actual cell swelling is induced in a dose dependent manner, as evidenced by progressively reduced cell density. Human RBC's tolerate flunarizine doses at least up to a concentration of $50\,mg\,l^{-1}$ without excessive cell swelling to the point of decrease in red cell deformability. Rat erythrocytes, on the other hand, are much smaller initially; consequently, an increase in cell volume of similar magnitude as in human cells will cause a proportionally larger change in the cell's surface area to volume ratio. It appears from our results that at a flunarizine concentration of $50\,mg\,l^{-1}$, the rat RBC's have swollen beyond optimal geometric proportions to a state which is no more deformable than the dense condition in a hypoxic environment. The morphological correlation is that the rat cells go beyond stomatocytic to more overtly spherical shape, a less deformable geometry.

Fenton and Sutherland (1992) have recently reported intertumoural differences in changes in oxygen delivery caused by flunarizine. Doses of flunarizine ($5\,mg\,kg^{-1}$) similar in magnitude to the lower concentrations tested in our experiments substantially improved oxygen delivery in KHT sarcomas rather than in RIF-1 tumours implanted subcutaneously in mice. Notably, the KHT tumours are more hypoxic initially than the RIF-1, as demonstrated in their control tumours by a lower percentage of blood vessels with at least 10% hemoglobin saturation. These results are entirely consistent with the mechanism of action demonstrated in our experiments. Flunarizine is unlikely to improve upon the regional blood flow within a tumour unless there is a reason (e.g. severe hypoxia) why the red blood cells might be less deformable locally within that region.

In summary, the results reported herein support the idea that the milieu of the hypoxic centres of solid tumours can produce loss of deformability of red blood cells. The drug flunarizine has activity in these circumstances which is characterised by stomatocytogenic changes in red cell morphology, a shift toward lower proportions of the less deformable high density ($>1.105\,g\,ml^{-1}$) red blood cells, and a consequent reduction in the viscosity of bulk suspensions of RBC's. Combined with the available data in animal models documenting enhanced radiosensitivity *in vivo* with flunarizine, this explanation of the mechanism of activity has encouraged us to pursue implementing the drug within the context of an investigational protocol in human cancer patients.

The authors wish to thank Jeri Edwards, Braven Beatty, and Ping Beall for invaluable expert assistance in the performance of all experiments. The work was supported in part by NIH/NCI grant RO1 CA40355 and National Heart, Lung, and Blood Institute grants HL 23728 and HL 28391.

### References

BESSIS, M. (1973). *Living Blood Cells and Their Ultrastructure*. Translated by Robert I. Weed. New York: Springer-Verlag.

BRERETON, W. & MURPHY, J.R. (1974). Rheologic studies of deoxygenated normal and hereditary spherocytosis blood and separated erythrocytes. *J. Lab. Clin. Med.*, **83**, 112–118.

BURNS, N.R. & GRATZER, W.B. (1985). Interaction of calmodulin with the red cell and its membrane skeleton and with spectrin. *Biochemistry*, **24**, 3070–3074.

CLARK, M., MOHANDAS, N., FEO, C., JACOBS, M.S. & SHOHET, S.B. (1981). Separate mechanisms of deformability loss in ATP-depleted and Ca-loaded erythrocytes. *J. Clin. Invest.*, **67**, 531–539.

CRANDALL, E.D., CRITZ, A.M., OSHER, A.S., KELJO, D.J. & FORSTER, R.E. (1978). Influence of pH on elastic deformability of the human erythrocyte membrane. *Am. J. Physiol.*, **235**, C269–C278.

DE CREE, J., DE COCK, W., GEUKENS, H., DE CLERCK, F., BEERENS, M. & VERHAEGEN, H. (1979). The rheological effects of cinnarizine and flunarizine in normal and pathologic conditions. *Angiology*, **30**, 505–515.

DEWHIRST, M.W., ONG, E.T., KLITZMAN, B., SECOMB, T.W., VINUYA, R.Z., DODGE, R., BRIZEL, D. & GROSS, J.F. (1992*a*). Pervascular oxygen tensions in a dorsal flap window chamber. *Radiat. Res.*, (in press).

DEWHIRST, M.W., ONG, E.T., MADWED, D., KLITZMAN, B., SECOMB, T., BRIZEL, D., BONAVENTURA, J., ROSNER, G., KAVANAGH, B., EDWARDS, J. & GROSS, J. (1992*b*). Effects of the calcium channel blocker flunarizine on tumor microvascular hemodynamics and oxygenation. *Radiat. Res.*, (in press).

FENTON, B.M. & SUTHERLAND, R.M. (1992). Effect of flunarizine on micro-regional distributions of intravascular $HbO_2$ saturations in RIF-1 and KHT sarcomas. *Int. J. Radiat. Oncol. Biol. Phys.*, **22**, 447–450.

FISHBEIN, W.N., DAVIS, J.I., FOELLMER, J.W. & CASEY, M.R. (1988). Clinical assay of the human erythrocyte lactate transporter. *Biochemical Med. Metabolic Biol.*, **39**, 351–359.

FLAMENG, W., VERHEYEN, F., BORGERS, M., DE CLERCK, F. & BRUGMANS, J. (1979). The effect of flunarizine treatment on human red blood cells. *Angiology*, **30**, 516–525.

FOXDAL, P., SJODIN, B., RUDSTAM, H., OSTMAN, C., OSTMAN, B. & HEDENSTIERNA, G.C. (1990). Lactate concentration differences in plasma, whole blood, capillary finger blood and erythrocytes during submaximal graded exercise in humans. *Eur. J. Appl. Physiol.*, **61**, 218–222.

FRANK, R.S. & HOCHMUTH, R.M. (1988). The influence of red cell mechanical properties on flow through single capillary-sized pores. *J. Biomech. Eng.*, **110**, 155–160.

GROSS, G.P. & HATHAWAY, W.E. (1972). Fetal erythrocyte deformability. *Pediatr. Res.*, **6**, 593–599.

GUEZENNEC, C.Y., NADAUD, J.F., SATABIN, P., LEGER, F. & LAFARGUE, P. (1989). Influence of polyunsaturated fatty acid diet on the hemorheological response to physical exercise in hypoxia. *Int. J. Sports Med.*, **10**, 286–291.

GULLINO, P.M., CLARK, S.H. & GRANTHAM, F.H. (1964). The interstitial fluid of solid tumors. *Cancer Res.*, **24**, 780–798.

HAKIM, T.S. & MACEK, A.S. (1988). Effect of hypoxia on erythrocyte deformability in different species. *Biorheology*, **25**, 857–868.

HERMANN, T., VASSELON, C., GEYSSANT, A. & HEALY, J.C. (1981). RBC filterability, oxygen saturation, ATP intracellular stock, and cerebral microcirculation. *Scand. J. Clin. Lab. Invest.*, **41** (suppl. 156), 213–216.

HEYKANTS, J., DE CREE, J. & HORIG, C. (1979). Steady-state plasma levels of flunarizine in chronically treated patients. *Arzneimittel-Forschung*, **29**, 1168–1171.

HORSMAN, M.R., CHAPLIN, D.J. & OVERGAARD, J. (1991). The use of blood flow modifiers to improve the treatment response of solid tumors. *Radiother. Oncol.*, **20** (Suppl.), 47–52.

JAIN, R.K. (1988). Determinants of tumor blood flow. *Cancer Res.*, **48**, 2641–2658.

KAELIN, W.G., SHRIVASTAV, S. & JIRTLE, R.L. (1984). Blood flow to primary tumors and lymph node metastases in SMT-2A tumor-bearing rats following intravenous flunarizine. *Cancer Res.*, **44**, 896–899.

KOUTSOURIS, D., DELATOUR-HANSS, E. & HANSS, M. (1985). Physico-chemical factors of erythrocyte deformability. *Biorheology*, **22**, 119–132.

LACELLE, P.L. & WEED, R.I. (1970). Low oxygen pressure: a cause of erythrocyte membrane rigidity. *J. Clin. Invest.*, **49**, 54a–55a.

LUGNIER, C., FOLLENIUS, A., GERARD, D. & STOCLET, J.C. (1984). Bepridil and flunarizine as calmodulin inhibitors. *Eur. J. Pharmacol.*, **98**, 157–158.

MEISELMAN, H.J. (1981). Morphological determinants of red cell deformability. *Scand. J. Clin. Lab. Invest.*, **41** (Suppl. 156), 27–34.

NELSON, G.A., ANDREWS, M.L. & KARNOVSKY, M.J. (1983). Control of erythrocyte shape by calmodulin. *J. Cell Biol.*, **96**, 730–735.

NOJI, S., TAKAHASHI, T. & KON, H. (1982). A spin-label study of the correlation between stomatocyte formation and membrane fluidization of erythrocytes. *Biochem. Pharmacol.*, **31**, 3173–3180.

NOJI, S., TANIGUCHI, S. & KON, H. (1987). Spin label study of erythrocyte deformability: $Ca^{2+}$-induced loss of deformability and the effects of stomatocytogenic reagents on the deformability loss in human erythrocytes in shear flow. *Biophys. J.*, **52**, 221–227.

NORTON, J.M., BARKER, N.D. & RAND, P.W. (1981). Effect of cell geometry, internal viscosity, and pH on erythrocyte filterability. *Proc. Soc. Exp. Biol. Med.*, **166**, 449–456.

RAND, P.W., AUSTIN, W.H., LACOMBE, E. & BARKER, N. (1968). pH and blood viscosity. *J. Appl. Physiol.*, **25**, 550–559.

SCHEUFLER, E., VOGELGESANG, R., WILFFERT, B., PEGRAM, B.L., HUNTER, J.B., WERMELSKIRCHEN, D. & PETERS, T. (1990). Uptake of catamphiphilic drugs into erythrocytic and muscular tissue correlates to membrane enrichment and to $^{45}Ca$ displacement from phosphatidylserine monolayers. *J. Pharmacol. Exp. Therapeutics*, **252**, 333–338.

SEVICK, E.M. & JAIN, R.K. (1991). Effect of RBC deformability on tumor blood flow: increase in viscous resistance during hyperglycemia. *Cancer Res.*, **51**, 2727–2730.

STONE, P.H., ANTMAN, E.M., MULLER, J.E. & BRAUNWALD, E. (1980). Calcium channel blocking agents in the treatment of cardiovascular disorders. *Ann. Int. Med.*, **93**, 886.

THOMAS, P.G., ZIMMERMAN, A.G. & VERKLEIJ, A.J. (1988). Prevention of calcium-induced membrane structural alterations in erythrocyte membranes by flunarizine. *Biochem. Biophys. Acta*, **946**, 439–444.

TRAN-SON-TAY, R., BEATTY, B.B., ACKER, D.N. & HOCHMUTH, R.M. (1988). Magnetically driven, acoustically tracked, translating-ball rheometer for small, opaque samples. *Rev. Sci. Instrum.*, **59**, 1399–1404.

TRAN-SON-TAY, R., COFFEY, B.E. & HOCHMUTH, R.M. (1989). A rheological study of packed red blood cell suspensions with an oscillating ball microrheometer. *Biorheology*, **26**, 143–151.

USAMI, S., CHIEN, S. & BERTLES, J.F. (1975). Deformability of sickle cells as studied by microsieving. *J. Lab. Clin. Med.*, **86**, 274–279.

VAUPEL, P., KALLINOWSKI, F. & OKUNIEFF, N. (1989). Blood flow, oxygen and nutrient supply, and metabolic microenvironment of human tumors: a review. *Cancer Res.*, **49**, 6449–6465.

VAUPEL, P. & MENKE, H. (1987). Blood flow, vascular resistance and oxygen availability in malignant tumours upon intravenous flunarizine. *Adv. Exp. Med. Biol.*, **215**, 393–398.

WALDENLIND, L., EDLUND, B.L., HULTING, J., KARNELL, J., LUND, F. & ROSENHAMMER, G. (1988). Decreased red cell filterability in patients with acute myocardial infarction. *Acta Med. Scandinavica*, **224**, 225–229.

WOOD, P.J. & HIRST, D.G. (1988). Cinnarizine and flunarizine as radiation sensitisers in two murine tumours. *Br. J. Cancer*, **58**, 742–745.

WOOD, P.J. & HIRST, D.G. (1989). Modification of tumour response by calcium antagonists in the SCVII/St tumour implanted at two different sites. *Int. J. Radiat. Biol.*, **56**, 355–367.

YARDIN, G. & MEISELMAN, H.J. (1989). Effects of cellular morphology on the viscoelastic behavior of high hematocrit RBC suspensions. *Biorheology*, **26**, 153–175.